# INTEGRATE II: randomised phase III controlled trials of regorafenib containing regimens versus standard of care in refractory Advanced Gastro-Oesophageal Cancer (AGOC): a study by the Australasian Gastro-Intestinal Trials Group (AGITG)

**DOI:** 10.1186/s12885-023-10642-7

**Published:** 2023-02-22

**Authors:** Lyn Ley Lam, Nick Pavlakis, Kohei Shitara, Katrin M. Sjoquist, Andrew J. Martin, Sonia Yip, Yoon-Koo Kang, Yung-Jue Bang, Li-Tzong Chen, Markus Moehler, Tanios Bekaii-Saab, Thierry Alcindor, Christopher J. O’Callaghan, Niall C. Tebbutt, Wendy Hague, Howard Chan, Sun Young Rha, Keun-Wook Lee, Val Gebski, Anthony Jaworski, John Zalcberg, Timothy Price, John Simes, David Goldstein

**Affiliations:** 1 NHMRC Clinical Trials Centre, University of Sydney, Camperdown, Sydney, Australia; 2 Royal North Shore Hospital, Sydney, Australia; 3 National Cancer Centre Hospital East, Chiba, Japan; 4 Asan Medical Centre, Seoul, South Korea; 5 Seoul National University College of Medicine, Seoul, South Korea; 6 College of Medicine, Kaohsiung Medical University, Kaohsiung, Taiwan; 7 University Medical Centre Mainz, Mainz, Germany; 8 Mayo Clinic Comprehensive Cancer Centre, Phoenix, USA; 9 McGill University Health Centre, Montreal, Canada; 10 Olivia Newton-John Cancer Wellness & Research Centre, Melbourne, Australia; 11 Calvary Mater Newcastle, Waratah, Australia; 12 Yonsei Cancer Centre, Yonsei University Health System, Seoul, South Korea; 13 Seoul National University Bundang Hospital, Seongnam, South Korea; 14 Monash University Melbourne, Melbourne, Australia; 15 The Queen Elizabeth Hospital, Adelaide, Australia; 16 Prince of Wales Hospital, Sydney, Australia

**Keywords:** Advanced gastro-oesophageal cancer, Regorafenib, Nivolumab, Tyrosine kinase inhibitor, Clinical trial

## Abstract

**Background:**

Advanced gastro-oesophageal cancer (AGOC) carries a poor prognosis. No standard of care treatment options are available after first and second-line therapies. Regorafenib is an oral multi-targeted tyrosine kinase inhibitor targeting angiogenic, stromal, and oncogenic receptor tyrosine kinases. Regorafenib 160 mg daily prolonged progression free survival compared to placebo (INTEGRATE, phase 2). Regorafenib 80 mg daily in combination with nivolumab 3 mg/kg showed promising objective response rates (REGONIVO).

**Methods/design:**

INTEGRATE II (INTEGRATE IIa and IIb) platform comprises two international phase III randomised controlled trials (RCT) with 2:1 randomisation in favor of experimental intervention. INTEGRATE IIa (double-blind) compares regorafenib 160 mg daily on days 1 to 21 of each 28-day cycle to placebo. INTEGRATE IIb (open label) compares REGONIVO, regorafenib 90 mg days 1 to 21 in combination with intravenous nivolumab 240 mg days 1 and 15 each 28-day cycle with investigator’s choice of chemotherapy (control). Treatment continues until disease progression or intolerable adverse events as per protocol. Eligible participants include adults with AGOC who have failed two or more lines of treatment. Stratification is by location of tumour (INTEGRATE IIa only), geographic region, prior VEGF inhibitor and prior immunotherapy use (INTEGRATE IIb only). Primary endpoint is overall survival. Secondary endpoints are progression free survival, objective response rate, quality of life, and safety. Tertiary/correlative objectives include biomarker and pharmacokinetic evaluation.

**Discussion:**

INTEGRATE II provides a platform to evaluate the clinical utility of regorafenib alone, as well as regorafenib in combination with nivolumab in treatment of participants with refractory AGOC.

**Trial registration:**

INTEGRATE IIa prospectively registered 1 April 2016 Australia New Zealand Clinical Trial Registry: ACTRN12616000420448 (ClinicalTrials.gov NCT02773524). INTEGRATE IIb prospectively registered 10 May 2021 ClinicalTrials.gov: NCT04879368.

**Supplementary Information:**

The online version contains supplementary material available at 10.1186/s12885-023-10642-7.

## Background

Gastro-oesophageal cancer is the seventh most common cancer globally with an estimated 500,000 new cases diagnosed annually [1]. It is the sixth leading cause of cancer mortality globally [1]. Despite current treatments, advanced gastro-oesophageal cancer (AGOC) carries a poor prognosis, with a median overall survival (OS) of less than 12 months [2].

Chemotherapy is a well-established first- and second-line treatment option for patients with AGOC. The combination of fluoropyrimidine and platinum-based chemotherapy is commonly used in the first line setting in patients with human epidermal growth factor receptor 2 (HER2) negative AGOC [3]. Trastuzumab is added to doublet chemotherapy in patients with HER2 positive AGOC [3]. First line chemotherapy in AGOC improves OS compared to best supportive care (HR 0.37, 95% CI 0.24–0.55), with longer OS seen with doublet chemotherapy compared to single agent chemotherapy (HR 0.82, 95% CI 0.74–0.90) [3]. In the second-line setting, commonly used regimens include chemotherapy (taxanes or irinotecan) [4–6] ramucirumab as monotherapy, and ramucirumab in combination with chemotherapy [7]. Second-line chemotherapy also improves OS in patients with AGOC compared to best supportive care (HR 0.73, 95% CI 0.58–0.96) [8]. In the third or later line setting, trifluridine/tipiracil improves median OS by 5.7 months compared to 3.6 months placebo in the TAGS trial (HR 0.69, 95% CI 0.56–0.85) [9]. In the phase III ATTRACTION-2 study, nivolumab improves median OS when compared to placebo in participants with AGOC who were refractory or intolerant to two or more lines of chemotherapy (HR 0.63, 95% CI 0.51–0.78) [10].

Regorafenib (BAY 73–4506) is a promising treatment option in AGOC. Regorafenib is an oral multikinase inhibitor (TKI) that targets angiogenic (VEGF, TIE-2), stromal (PDGF-β), and oncogenic (RAF, RET and KIT) receptor tyrosine kinases [11]. Regorafenib showed promise in the treatment of participants with chemotherapy refractory AGOC in the INTEGRATE trial [12]. INTEGRATE was an international phase II multi-centre Randomised Controlled Trial (RCT), sponsored by the Australasian Gastro-Intestinal Trials Group (AGITG), comprising of 152 participants with AGOC who had progressed on first- and/or second-line chemotherapy [12]. INTEGRATE demonstrated an improved progression free survival (PFS) with regorafenib at a dose of 160 mg daily on days 1 to 21 of a 28-day cycle compared to placebo (HR 0.40, 95% CI 0.28–0.59, *p* < 0.001) [12]. There was a trend towards an OS benefit with regorafenib (HR 0.74, 95% CI 0.51–1.08, *p* = 0.147), but the study was underpowered to reliably detect a plausible OS effect and 58% of placebo participants crossed over to receive regorafenib following disease progression [12]. Pre-specified analyses demonstrated a higher efficacy of regorafenib amongst participants in Korea compared to participants in Australia, New Zealand, and Canada (HR 0.12 vs HR 0.61, *p* < 0.001) suggesting a difference in progression-free survival by ethnicity/geographical region [8]. There were no unexpected toxicities with regorafenib [12].

The results of INTEGRATE formed the basis for launching the original INTEGRATE II phase III RCT with the aim of investigating whether regorafenib at a dose of 160 mg daily given on days 1 to 21 of a 28-day cycle is better than placebo in prolonging OS in participants with refractory AGOC who have failed two or more lines of chemotherapy. Both arms received best supportive care and crossover of placebo participants to receive regorafenib at disease progression was not allowed.

After INTEGRATE II opened for recruitment, standard practice in the management of AGOC shifted with increasing evidence through phase II [13, 14] and phase III RCTs supporting the use of immune checkpoint inhibitors in first and later line treatment [10, 15]. For example, the combination of nivolumab plus chemotherapy in CheckMate 649 resulted in a significant improvement in OS (HR 0.71, 98% CI 0.59–0.86, *p* < 0.0001) and PFS (HR 0.68, 98% CI 0.56–0.81, *p* < 0.0001) in participants with AGOC and a PD-L1 combined positive score (CPS) score of 5 or more in the first line setting [15]. Although the combination of upfront chemotherapy and immune checkpoint inhibitors is suggested to be superior to chemotherapy alone in the first line treatment of AGOC [15], most patients will eventually experience disease progression despite aggressive upfront therapies. This highlighted the need for the evaluation of newer combination therapies to overcome primary and acquired resistance to current therapies.

The INTEGRATE II research plan was consequently expanded to become a platform protocol that included a new second trial comparing regorafenib in combination with nivolumab (experimental treatment) against chemotherapy (control treatment). This new study was called INTEGRATE IIb and the original study was renamed INTEGRATE IIa.

INTEGRATE IIb is a phase III RCT that investigates whether regorafenib 90 mg daily on day 1 to 21 of a 28-day cycle in combination with nivolumab 240 mg every 2 weeks is superior to chemotherapy alone in prolonging OS in the overall and Asian sub-population of participants with AGOC who have progressed following two or more lines of treatment. The rationale behind INTEGRATE IIb is the REGONIVO phase Ib trial, a dose-finding/dose expansion Japanese study which assessed the combination of regorafenib and nivolumab in 50 participants with previously treated gastric and colorectal cancers [16]. As part of the dose expansion study, regorafenib was given at a dose of 80 mg to 160 mg once daily on days 1 to 21 of a 28-day cycle together with nivolumab 3 mg/kg every 2 weeks until disease progression or dose limiting toxicities [16]. The authors of REGONIVO concluded that the optimal dose of regorafenib when given in combination with nivolumab 3 mg/kg every 2 weeks was 80 mg daily on days 1 to 21 of a 28-day cycle [16]. The overall response rate (ORR) in the REGONIVO study was 44% in microsatellite stable (MSS) gastric cancer and 33% in MSS colorectal cancer [16]. These impressive ORR results provided a rationale for further investigation of this combination therapy in larger cohorts [16].

INTEGRATE IIa and INTEGRATE IIb are two relevant trials that will further clarify the role of regorafenib in the treatment of AGOC. INTEGRATE IIa will answer the important question of whether regorafenib monotherapy is better than placebo in participants with chemotherapy refractory AGOC. INTEGRATE IIa will also provide information on the role of regorafenib monotherapy in participants who cannot have further chemotherapy or immune checkpoint inhibitors. INTEGRATE IIb seeks to evaluate the efficacy of regorafenib in combination with immune checkpoint inhibitors in comparison to standard of care chemotherapy in the third or later line setting.

## Methods/ Design

### Aim

The aim of INTEGRATE IIa is to determine whether regorafenib160mg daily on day 1 to 21 of a 28-day cycle improves OS compared to placebo. The aim of INTEGRATE IIb is to determine whether regorafenib 90 mg daily on day 1 to 21 of a 28-day cycle in combination with nivolumab 240 mg every 2 weeks improves OS compared to investigator’s choice of chemotherapy in participants with AGOC who have progressed or were intolerant to two or more lines of chemotherapies including platinum and fluoropyrimidine based regimens. After 2 months, nivolumab can be given monthly at a dose of 480 mg.

### Design

**Fig. 1:**
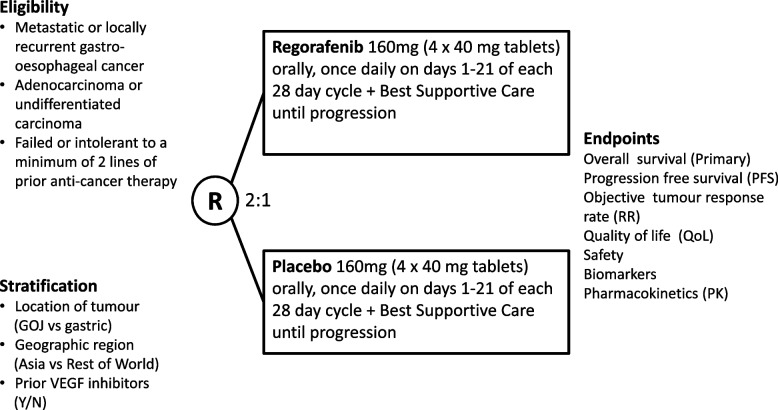
Schema of INTEGRATE IIa

INTEGRATE IIa is a prospective, multi-centre, comparative, double-blinded phase III RCT where participants with histological or cytological confirmation of AGOC or undifferentiated carcinoma of primary gastro-oesophageal origin are randomised 2:1 to receive either regorafenib monotherapy (Arm A) or placebo (Arm B) (Fig. 1). Randomisation is stratified by location of tumour (GOJ (gastro-oesophageal junction) vs. gastric), geographic region (Asia vs. rest of world) and prior VEGF inhibitors (yes vs no).

**Fig. 2:**
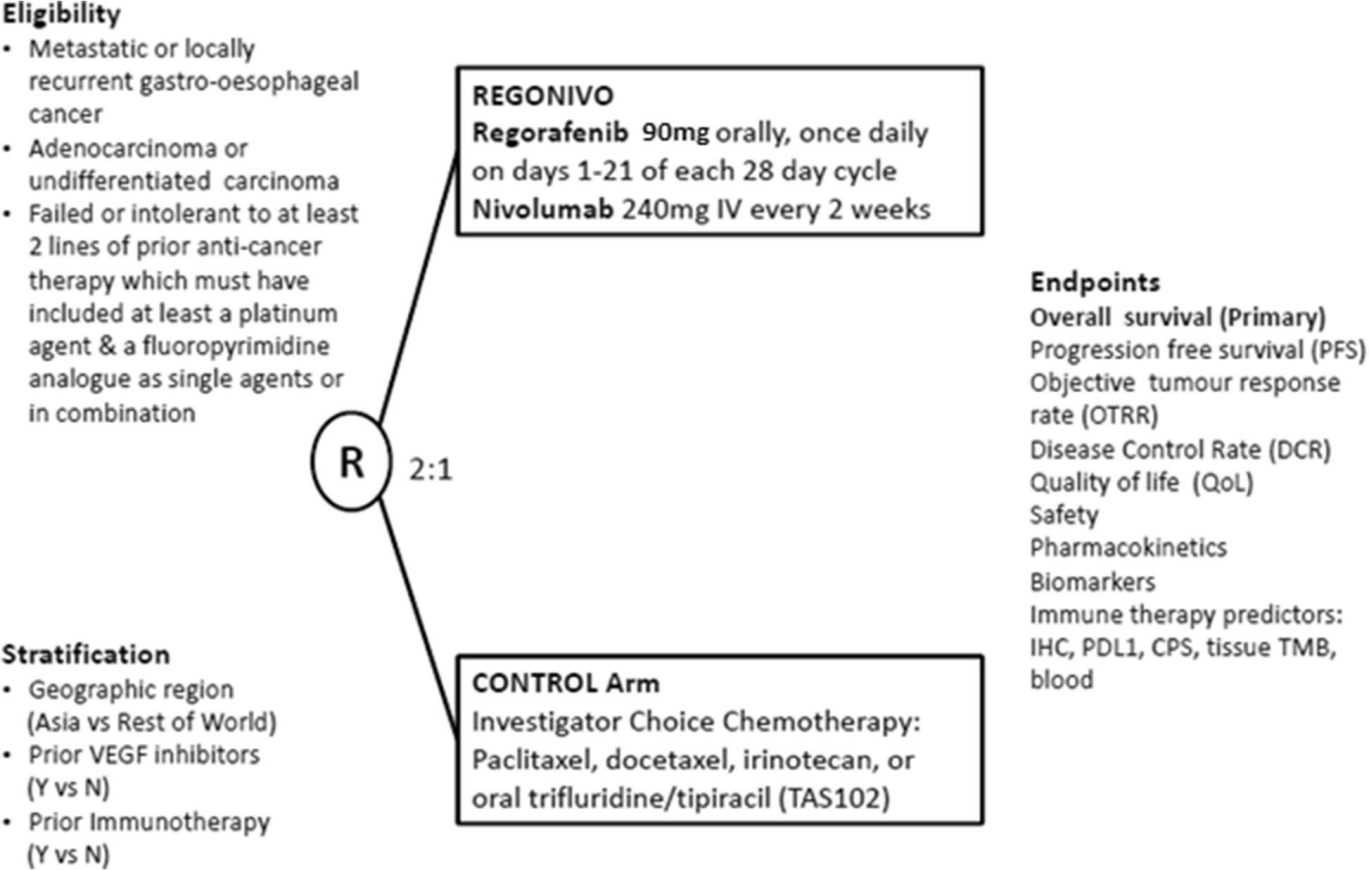
Schema of INTEGRATE IIb

INTEGRATE IIb is a prospective, multi-centre, comparative, open label phase III RCT where participants with histological or cytological confirmation of AGOC or undifferentiated carcinoma of primary gastro-oesophageal origin are randomised 2:1 to receive regorafenib and nivolumab (experimental arm) or investigator’s choice of chemotherapy (control arm) (Fig. 2). Randomisation is stratified by geographic region (Asia vs. rest of world), prior VEGF inhibitors (yes vs no) and prior immunotherapy (yes vs no).

For INTEGRATE IIa and INTEGRATE IIb, all treatments are to be administered until disease progression, unacceptable toxicity, withdrawal of consent, more than 2 dose reductions due to treatment related adverse events or day 1 treatment delays of longer than 28 days. Randomisation will be performed centrally. Participant quality of life (QOL) questionnaires are to be completed on cycle 1 day 1 of treatment and every 4 weeks thereafter during treatment and at the time of disease progression. Participants who end treatment before disease progression will have QOL data collected every 8 weeks until disease progression. A list of participating centres is provided in Table 1 for INTEGRATE IIa and Table 2 for INTEGRATE IIb.

**Table 1:** List of Participating Centres for INTEGRATE IIa

Site Name	Principal Investigator
Canberra Hospital	Dr Desmond Yip
Royal North Shore Hospital	Prof Nick Pavlakis
Westmead Hospital	Dr Adnan Nagrial
Prince of Wales Hospital	Prof David Goldstein
Concord Repatriation General Hospital	Dr Philip Beale
St Vincent's Public Hospital	Dr Hao-Wen Sim
St George Hospital	Dr Katrin Sjoquist
Coffs Harbour Health Campus	Dr Karen Briscoe
Gosford Hospital	Dr Matthew Wong
The Tweed Hospital	Dr Sumit Lumba
Port Macquarie Base Hospital	Dr Stephen Begbie
Newcastle Private Hospital	Dr Antonino Bonaventura
Austin Hospital	A/Prof Niall Tebbutt
Border Medical Oncology	Dr Craig Underhill
Ballarat Oncology and Haematology Services	Prof George Kannourakis
Monash Medical Centre	Dr Andrew Strickland
Royal Brisbane and Womens Hospital	Dr Matthew Burge
Townsville Hospital	Dr Suresh Varma
Sunshine Coast University Hospital	Dr Alessandra Francesconi
Flinders Medical Centre	A/Prof Chris Karapetis
Ashford Cancer Centre Research	Dr Carolyn Bampton
The Queen Elizabeth Hospital	Prof Tim Price
Sir Charles Gairdner Hospital	Dr Kevin Jasas
St John of God Hospital Subiaco	Dr Tom van Hagen
Royal Hobart Hospital	Dr Louise Nott
Royal Darwin Hospital	Dr Narayan Karanth
Auckland Hospital	Prof Michael Findlay
Seoul National University Hospital	Dr Do Youn Oh
Asan Medical Centre	Dr. Yoon Koo Kang
Samsung Medical Center	Dr Jeeyun Lee
Yonsei University Health System – Severance Hospital	Dr Sun Young Rha
Seoul National University Bundang Hospital	Dr Keun-Wook Lee
Korea University Anam Hospital	Dr Yeul Hong Kim
Yonsei University Health System – Gangnam Severance Hospital	Dr Jae Yong Cho
The Catholic University of Korea – Yeouido St. Mary’s Hospital	Dr In Sook Woo
The Catholic University of Korea – Seoul St. Mary’s Hospital	Dr Sang Young Roh
SMG-SNU Boramae Medical Center	Dr Jin-Soo Kim
Hallym University Sacred Heart Hospital	Dr Dae Young Zang
Chonbuk National University Hospital	Dr Jin-Soo Kim
Korea University Guro Hospital	Dr Sang-Cheul Oh
Chungbuk National University Hospital	Dr Hye Sook Han
Chung-Ang University Hospital	Dr In Gyu Hwang
Gyeongsang University Hospital	Dr Jung Hun Kang
Dong-A University Hospital	Dr Sung Yong Oh
Haeundae Paik Hospital	Dr. Sung-Sook Lee
Kangbuk Samsung Hospital	Dr. Dong-Hoe Koo
National Taiwan University Hospital (NTUH)	Dr. Kun-Huei Yeh
Taipei Veterans General Hospital (TPVGH)	Dr. Yee Chao
National Cheng Kung University Hospital (NCKUH)	Dr. Li-Tzong Chen
Kaohsiung Medical University Chung-Ho Memorial Hospital	Dr. Jaw-Yuan Wang
China Medical University Hospital (CMUH)	Dr. Li-Yuan Bai
National Cancer Centre Hospital East	Dr. Kohei Shitara
Hokkaido University Hospital	Dr. Yoshito Komatsu
Kyushu Cancer Centre	Dr. Taito Esaki
Shizuoka Cancer Centre	Dr. Nozomu Machida
Saitama Cancer Centre	Dr. Hiroki Hara
QEII Health Sciences Centre Capital District Health Authority	Dr. Stephanie Snow
The Research Institute of the McGill University Health Centre	Dr. Thierry Alcindor
Ottawa Hospital Research Institute	Dr. Rachel Goodwin
Jewish General Hospital	Dr. Petr Kavan
USC Norris	Dr. Syma Iqbal
Mayo Clinic Arizona	Dr. Tanios Bekaii-Saab

**Table 2:** List of Participating Centres for INTEGRATE IIb

Site Name	Principal Investigator
Royal North Shore Hospital	Prof Nick Pavlakis
Westmead Hospital	Dr Adnan Nagrial
Prince of Wales Hospital	A/Prof David Goldstein
Concord Repat General Hospital	Dr Philip Beale
St Vincent’s Public Hospital	Dr Hao Wen Sim
St George Hospital	Dr Katrin Sjoquist
Coffs Harbour Health Campus	Dr Karen Briscoe
Gosford Hospital	Dr Matthew Wong
The Tweed Hospital	Dr Sumit Lumba
Port Macquarie Base Hospital	Dr Stephen Begbie
Newcastle Private Hospital	Dr Antonio Bonaventura
Austin Hospital	A/Prof Niall Tebbutt
Border Medical Oncology Research Unit	Dr Craig Underhill
Ballarat Oncological & Haematological Services	Prof George Kannourakis
Monash Medical Centre	Dr Andrew Strickland
Royal Brisbane & Women’s Hospital	Dr Matthew Burge
Townsville Hospital	Dr Suresh Varma
Sunshine Coast University Hospital	Dr Alessandra Francesconi
Flinders Medical Centre	A/Prof Chris Karapetis
The Queen Elizabeth Hospital	Prof Tim Price
Sir Charles Gairdner Hospital	Dr Kevin Jasas
St John of God Hospital (Subiaco)	Dr Tom van Hagen
Royal Hobart Hospital	Dr Louise Nott
Royal Darwin Hospital	Dr Narayan Karanth
Shikoku Cancer Center	Dr Tomohiro Nishina
National Cancer Center Hospital East	Dr. Kohei Shitara
Hokkaido University Hospital	Dr. Yoshito Komatsu
Kyushu Cancer Center	Dr Taito Esaki
Shizuoka Cancer Center	Dr. Nozomu Machida
Saitama Cancer Center	Dr. Hiroki Hara
Seoul National University Hospital	Dr Do Youn Oh
Asan Medical Centre	Dr. Yoon Koo Kang
Yonsei University Health System – Severance Hospital	Dr Sun Young Rha
Seoul National University Bundang Hospital	Dr Keun-Wook Lee
Korea University Anam Hospital	Dr Yeul Hong Kim
Yonsei Uni Health System – Gangnam Severance Hospital	Dr Jae Yong Cho
The Catholic Uni of Korea – Yeouido St. Mary’s Hospital	Dr In Sook Woo
The Catholic Uni of Korea – Seoul St. Mary’s Hospital	Dr Sang Young Roh
SMG-SNU Boramae Medical Center	Dr Jin-Soo Kim
Hallym University Sacred Heart Hospital	Dr Dae Young Zang
Jeonbuk National University Hospital	Dr Jin-Soo Kim
Korea University Guro Hospital	Dr Sang-Cheul Oh
Chungbuk National University Hospital	Dr Hye Sook Han
Chung-Ang University Hospital	Dr In Gyu Hwang
Gyeongsang University Hospital	Dr Jung Hun Kang
Dong-A University Hospital	Dr Sung Yong Oh
Kangbuk Samsung Hospital	Dr. Dong-Hoe Koo
Haeundae Paik Hospital	Dr. Sung-Sook Lee
National Taiwan University Hospital (NTUH)	Dr. Kun-Huei Yeh
Taipei Veterans General Hospital (TPVGH)	Dr. Yee Chao
National Cheng Kung University Hospital (NCKUH)	Dr. Li-Tzong Chen
Kaohsiung Medical Uni Chung-Ho Memorial Hospital	Dr. Jaw-Yuan Wang
China Medical University Hospital (CMUH)	Dr. Li-Yuan Bai
USC Norris	Dr. Syma Iqbal
Mayo Clinic Arizona	Dr Tanios Bekaii-Saab
Illinois CancerCare—Peoria	Dr Jijun Liu
Lexington Health Inc	Dr James Wells
Monument Health Rapid City Hospital	Dr Abdel-Ghani Azzouqa
Siouxland Regional Cancer Center	Dr Donald Wender
University of Washington Medical Center	Dr David Zhen
Klinikum rechts der Isar der TU München	Prof. Volker Heinemann
Universitätsklinikum Leipzig	Dr Gertraud Stocker
Studienzentrum Onkologie Ravensburg	Prof. Tobias Dechow
Institut für Klinisch Onkol Forschung am Krankenhaus Nordwest	Dr Thorsten Goetze
Universitätsklinikum Jena	Dr Med. Udo Lindig
Universitätsklinikum Mainz	Prof. Markus Möhler
Philipps-Universitat Marburg	Dr Jorge Riera Knorrenschild
Universitätsklinikum Heidelberg	Dr Georg Martin Haag
Helios Bad Saarow	Dr Daniel Pink
Klinikum Bayreuth	Prof. Alexander Kiani
KEM/Evang. Kliniken Essen Mitte gGmbH	Dr Christian Müller
Norddeutsches Studienzentrum für Innovative Onkologie (NIO)	Dr Eray Gökkurt
Charité Universitätsmedizin Berlin	Dr Peter Thuss-Patience
Klinikum Magdeburg gGmbH	Dr Jorge Riera Knorrenschild
Caritas Klinikum Saarbrücken St. Theresia	Prof. Manfred P. Lutz
Klinikum Leverkusen gGmbH	Dr Andrea Heider
Universitätsklinikum Ulm	Dr Thomas Ettrich
Evang. Klinikum Bethel Bielefeld	Dr Kambiz Taghizadeh
Universitätsklinikum Bonn	Dr Maria Gonzalez-Carmona
Kliniken der Stadt Köln	Dr. Stefan Angermeier
Universitätsklinikum Greifswald	Prof. Ali Aghdassi
Klinikum Ludwigburg	Dr. Bernhard Sibbing

### Study endpoints

The primary end point for INTEGRATE IIa and INTEGRATE IIb is overall survival in each arm, defined as the interval from date of randomisation to date of death from any cause or the date the patient was last known to be alive. Secondary objectives for INTEGRATE IIa and INTEGRATE IIb are PFS, ORR (according to RECIST v1.1), QOL and safety profile (rates of adverse events per CTCAE v4.03). The tertiary and correlative endpoints may include: investigation of VEGF-related biomarkers and biomarkers relating to angiogenesis and/or tumourigenesis in blood and tumour as prognostic and/or predictive markers for study endpoints relating to survival, response and safety; regorafenib levels to evaluate pharmacokinetics in participants from different geographical regions; evaluation of the prevalence and distribution of the four proposed molecular phenotypes of gastric cancer proposed by the Cancer Genome Atlas Research Network [17], and their association with angiogenic biomarkers and regorafenib activity. Additional correlative endpoints for INTEGRATE IIb may include associations between autoimmunity and clinical outcomes, immune profiling of tumour, tumour mutational burden and cellular and molecular signatures associated with immune related adverse events.

### Study population

INTEGRATE IIa and INTEGRATE IIb have similar eligibility criteria:

Participants aged ≥ 18 years with ECOG performance status < 2, who have histological or cytological confirmation of metastatic or locally advanced gastro-oesophageal carcinoma (gastric or GOJ cancer). Participants must have failed or be intolerant to a minimum of 2 lines of prior anti-cancer therapy and must have had at least one platinum agent and one fluoropyrimidine analogue.

Other main eligibility criteria include:

- 1. Evaluable disease according to RECIST 1.1
- 2. Adenocarcinoma or undifferentiated carcinoma histology
- 3. Neoadjuvant or adjuvant chemotherapy or chemoradiotherapy will be considered as first line treatment if the patient has relapsed or progressed within 6 months of completing treatment
- 4. Ramucirumab monotherapy or immunotherapy with a checkpoint inhibitor will be considered a line of treatment
- 5. HER2 positive participants must have previously received trastuzumab
- 6. Adequate organ function defined as below:
  - i. Bone marrow: ANC (absolute neutrophil count) ≥ 1500/μl, platelets ≥ 100,000/μl, haemoglobin ≥ 9 g/dl. INR (international normalised ratio) and APTT (activated partial thromboplastin time) ≤ 1.5 × ULN (upper limit of normal). Note: participants previously on long-term anticoagulation with warfarin or low molecular weight heparin are eligible.
  - ii. Adequate liver function: Total bilirubin ≤ 1.5 × ULN; AST (aspartate transaminase), ALT (alanine transaminase) and/or ALP (alkaline phosphatase) ≤ 5 × ULN.
  - iii. Adequate renal function, creatinine clearance, as measured by the Cockcroft and Gault formula of > 50mls/minute.
  - iv. Adequate cardiac function: left ventricular ejection fraction ≥ 50% (INTEGRATE IIa only).
- 7. Study treatment to start within 7 days of randomisation

Main exclusion criteria include:

- 1. Poorly controlled hypertension (systolic blood pressure > 140 mmHg or diastolic pressure > 90 mmHg despite optimal medical management)
- 2. Prior exposure to anti-VEGF targeted therapy using small molecule VEGF TKIs. Prior anti-VEGF targeted monoclonal antibody therapies such as bevacizumab and ramucirumab are permitted
- 3. Any prior use of more than one immune checkpoint inhibitor (INTEGRATE IIb only)
- 4. Palliative radiotherapy unless more than 14 days between completion of radiation and date of registration and adverse events resulting from radiation have resolved to less than grade 2 according to CTCAE V4.03
- 5. Interstitial lung disease with ongoing signs and symptoms
- 6. Uncontrolled metastatic disease to central nervous system

### Study interventions

In INTEGRATE IIa, participants enrolled in Arm A will receive regorafenib 160 mg (4 × 40 mg tablets) orally once daily on days 1 to 21 of a 28-day cycle until disease progression, unacceptable toxicities, day 1 treatment delay of more than 28 days or withdrawal of consent. Participants enrolled in Arm B will receive matching placebo. Missed or vomited tablets cannot be compensated for by treatment later.

In INTEGRATE IIb, participants enrolled in the experimental arm of the study will receive regorafenib and nivolumab. Regorafenib will be self-administered orally once daily at a dose of 90 mg (3 × 30 mg tablets) on days 1 to 21 of a 28-day cycle until disease progression, unacceptable toxicities, day 1 treatment delay of more than 28 days or withdrawal of consent. Only one dose level reduction of regorafenib is permitted (90 mg to 60 mg) for management of regorafenib related adverse events. If regorafenib is not tolerated at a dose of 60 mg, regorafenib will be discontinued. Nivolumab will be administered intravenously at a dose of 240 mg on day 1 of a 14-day cycle until disease progression or prohibitive adverse events as per protocol or up to 24 infusions or 2 years maximum treatment duration. After 2 months, nivolumab can be administered at a dose of 480 mg on day 1 of a 28-day cycle. If a delay in nivolumab administration is required when a treatment cycle has commenced, nivolumab cannot be delayed more than 12 days for the 14-day schedule, and not more than 20 days for the 28-day schedule. If nivolumab needs to be delayed more than 12 weeks from last treatment date due to toxicity, nivolumab will be discontinued permanently. The continuation of regorafenib monotherapy is permitted despite permanent discontinuation of nivolumab. Participants enrolled in the control arm will receive investigator’s choice of chemotherapy with either docetaxel 60-75 mg/m^2^ intravenously every 21 days, or paclitaxel 135-250 mg/m^2^ intravenously on day 1 every 21 days, or paclitaxel 80 mg/m^2^ intravenously on days 1, 8, 15, 21 every 28 days, or irinotecan 250-300 mg/m^2^ intravenously on day 1 every 21 days, or irinotecan 150-180 mg/m^2^ intravenously on days 1 and 15 every 28 days, or irinotecan 125 mg/m^2^ intravenously on days 1 and 8 every 28 days, or trifluridine and tipiracil 35 mg/m^2^ up to 80 mg dose orally twice daily on days 1 to 5 and 8 to 12 of a 28 day cycle.

All participants in INTEGRATE IIa and INTEGRATE IIb will receive best supportive care which includes any intervention to preserve comfort and dignity such as stent insertion for gastric outlet obstruction, radiation to a bleeding primary tumour and use of any concomitant medications considered necessary for participant’s wellbeing including antiemetics, antidiarrhoeals, anti-inflammatory agents and analgesics.

### Study assessments

Disease assessment by CT scan will be performed at baseline and every 8 weeks, regardless of delays in drug administration until disease progression. QOL questionnaire (European Organization for Research and Treatment of Cancer (EORTC) QLQ-C30 and EORTC QLQ-STO22) will be completed at baseline and every 4 weeks during treatment and at the time of disease progression. For participants who end treatment before disease progression, QOL questionnaire will be collected every 8 weeks during follow up until disease progression. Tumour marker CA19-9 is assessed at the same time as CT imaging until disease progression.

### Translational research

Blood for biomarker research is collected from all participants at four timepoints: Cycle 1 Day 1, Cycle 2 Day 1, Cycle 4 Day 1, and end of treatment. Blood for pharmacokinetics (PK) is collected at three timepoints: Cycle 1 Day 15, Cycle 2 Day 1, and Cycle 2 Day 15 (optional) drawn before regorafenib is taken and 1 to 4 h afterwards. All blood samples collected are processed, frozen and biobanked. Archival tissue from the primary tumour or metastases are retrieved for translational research.

### Statistical considerations

The primary analysis for INTEGRATE IIa and IIb is a comparison of OS between the experimental and control arms using a log-rank test accounting for stratification factors. A HR for OS will be estimated from a stratified Cox proportional hazards model accounting for stratification factors. PFS will be analysed in a comparable fashion to OS. ORR will be compared using Cochran-Mantel–Haenszel test accounting for stratification factors or Fisher’s exact test if expected cell counts are low. Quality of life will be analysed using the approaches applied to the INTEGRATE trial [18]. A series of subgroup analyses will be performed on OS for the stratification factors used at randomisation.

When the INTEGRATE platform protocol was proposed, the original sample size target for INTEGRATE II (regorafenib vs placebo) of *N* = 350 was changed to *N* = 250 for the INTEGRATE IIa trial. A study with 250 participants randomised in a 2:1 ratio (regorafenib: placebo) to yield an expected 221 OS events and have 80% power to detect a HR for OS of 0.67 with a 2-sided α of 5%. The OS events from INTEGRATE IIa will also contribute to a pooled analysis with the previous INTEGRATE trial which had 123 OS events. The conditional power of this combined pooled analysis will have at least 90% power to detect a HR for OS of 0.67. To determine the appropriateness of a pooled analysis, heterogeneity of treatment effect on OS across INTEGRATE and INTEGRATE IIa will be tested. Assuming there is no statistically significant heterogeneity, a sequential closed testing procedure (with a 2-sided α of 5%) will be applied to the following null hypotheses of no treatment effect in the:

• Pooled cohort [INTEGRATE + INTEGRATE IIa] (${\mathrm{H}0}_{1}$)
• INTEGRATE IIa cohort (${\mathrm{H}0}_{2}$)
• Asian region pooled cohort [INTEGRATE + INTEGRATE IIa] (${\mathrm{H}0}_{3}$)
• Asian region INTEGRATE IIa cohort (${\mathrm{H}0}_{4}$)

For INTEGRATE IIb, a sample of 450 participants will be randomised 2:1 (regorafenib and nivolumab: chemotherapy) and followed until 380 deaths occur to provide at least 90% power to detect a HR for OS of 0.70 with a 2-sided α of 0.05. The design also accommodates early stopping for benefit/harm at an interim analysis performed at 2/3 of the required events using the error spending approach of Lan-DeMets. A safety analysis will be undertaken when a minimum of 8 participants are enrolled from Japan, 16 from Asia and the remainder from USA, Europe and Australasia. The final analysis on OS will involve testing the following two null hypotheses: (1) no treatment effect on OS in the whole trial cohort (${H}_{0}^{All}$); and, (2) no treatment effect on OS in the Asian region cohort (${H}_{0}^{Asian}$). A sequenced closed testing procedure will be used to constrain the overall type I error of these two tests to 5%.

### Safety

All adverse events for INTEGRATE IIa and INTEGRATE IIb will be recorded from the first dose of study treatment until 30 days (90 days for nivolumab) following last treatment dose. The investigator is responsible for ensuring all adverse events observed by the investigator or reported by the trial participants are documented in electronic case report forms (eCRFs). Serious adverse events (SAEs), including suspected unexpected serious adverse reactions (SUSAR) occurring during the study must be reported to the sponsor within 24 h of investigational site staff becoming aware of the event according to local procedures. The sponsor is responsible for the medical review of all SAEs and for their notification to the appropriate ethics committees and local authorities.

## Discussion

INTEGRATE IIa and INTEGRATE IIb address the clinically important question of exploring later line therapies that can improve overall survival in participants with refractory AGOC. INTEGRATE IIa and INTEGRATE IIb have similar inclusion/exclusion criteria and primary/secondary endpoints. The use of one previous line of immune checkpoint inhibitor is allowed in INTEGRATE IIb. The rationale for this is that some participants may have had immunotherapy in combination with chemotherapy as first line treatment for their AGOC as per the CheckMate 649 study [15]. Another rationale for this is that the combination of regorafenib and nivolumab in the REGONIVO study showed promising objective response rates [16].

There are a few major differences between INTEGRATE IIa and INTEGRATE IIb. INTEGRATE IIa compares regorafenib to placebo whilst INTEGRATE IIb compares regorafenib and nivolumab to chemotherapy. INTEGRATE IIa uses placebo as the control arm as there was minimal evidence at the time of protocol development to support third line chemotherapy as the standard of care. However, a systematic review and meta-analysis by Zheng et al. from 19 studies subsequently showed that third-line chemotherapy compared to placebo was associated with a better median OS (HR 0.68, 95% CI 0.57–0.82, *p* < 0.001) and progression-free survival (HR 0.56, 95% CI 0.44–0.71, *p* > 0.01) in participants with AGOC [19]. A randomised phase 3 study of trifluridine/tipiracil versus placebo also demonstrated an OS advantage in this pre-treated population (HR 0.69, 95% CI 0.56–0.85), *p* < 0.001) [9]. As such, it was decided that INTEGRATE IIb should use chemotherapy as the control arm.

Another difference between INTEGRATE IIa and INTEGRATE IIb is the starting dose of regorafenib. In INTEGRATE IIa, the starting dose of regorafenib is 160 mg (4 × 40 mg) whilst the starting dose of regorafenib is lower in INTEGRATE IIb 90 mg (3 × 30 mg). In the REGONIVO phase I study, the optimal dose of regorafenib when combined with nivolumab in terms of efficacy and safety is 80 mg [16]. However, since completion of the REGONIVO study, Bayer has developed a 30 mg tablet formulation dose for regorafenib that is expected to provide very similar exposure to 80 mg due to PK variability and will permit a dose reduction to 60 mg using 2 × 30 mg tablets dose level -1 = 33% reduction [20].

In summary, INTEGRATE II provides a platform to evaluate the clinical utility of regorafenib alone, as well as regorafenib in combination with nivolumab in the treatment of participants with AGOC who have failed two or more lines of treatment. The results of this study will hopefully improve the current standard of care in the treatment of AGOC.

## Supplementary Information

**Additional file 1:** Spirit Checklist.

## Data Availability

Not applicable.

## Abbreviations

AGOC: Advanced gastro-oesophageal cancer
HER2: Human epidermal growth factor receptor 2
TKI: Tyrosine kinase inhibitor
OS: Overall survival
PFS: Progression free survival
QOL: Quality of life
ORR: Objective response rate
CPS: Combined positive score
PK: Pharmacokinetics
ALP: Alkaline phosphatase
ANC: Absolute neutrophil count
APTT: Activated partial thromboplastin time
AST: Aspartate transaminase (or aspartate aminotransferase)
CTCAE: Common terminology criteria for adverse events
RECIST: Response evaluation criteria in solid tumours
ULN: Upper limit of normal
VEGF: Vascular endothelial growth factor
TIE-2: Tyrosine kinase with immunoglobulin and EGF homology domains
PDGF-β: Platelet derived growth factor receptor—β
RAF: Rapidly accelerated fibrosarcoma (raf) kinase
RET: Ret proto-oncogene
KIT: Proto-oncogene c-KIT
IDSMC: Independent data and safety monitoring committee
eCRFs: Electronic case report forms
HR: Hazard ratio
SAEs: Serious adverse events
SUSAR: Suspected unexpected serious adverse events
NHMRC: National health and medical research council clinical trials centre

## References

[1] Bray F, Ferlay J, Soerjomataram I, Siegel RL, Torre LA, Jemal A (2018). Global cancer statistics 2018: GLOBOCAN estimates of incidence and mortality worldwide for 36 cancers in 185 countries. CA A Cancer J Clin.

[2] Abbas MN, Bright T, Price T, Karapetis C, Thompson S, Connell C, Watson D, Barnes M, Bull J, Singhal N, Roy A (2021). Patterns of care and outcomes for gastric and gastro-oesophageal junction cancer in an Australian population. ANZ J Surg.

[3] Bang YJ, Van Cutsem E, Feyereislova A, Chung HC, Shen L, Sawaki A, Lordick F, Ohtsu A, Omuro Y, Satoh T, Aprile G, Kulikov E, Hill J, Lehle M, Rüschoff J, Kang YK (2010). ToGA Trial Investigators. Trastuzumab in combination with chemotherapy versus chemotherapy alone for treatment of HER2-positive advanced gastric or gastro-oesophageal junction cancer (ToGA): a phase 3, open-label, randomised controlled trial. Lancet.

[4] Lee KW, Maeng CH, Kim TY, Zang DY, Kim YH, Hwang IG, Oh SC, Chung JS, Song HS, Kim JW, Jeong SJ, Cho JY (2019). A Phase III Study to Compare the Efficacy and Safety of Paclitaxel Versus Irinotecan in Patients with Metastatic or Recurrent Gastric Cancer Who Failed in First-line Therapy (KCSG ST10-01). Oncologist.

[5] Hironaka S, Ueda S, Yasui H, Nishina T, Tsuda M, Tsumura T, Sugimoto N, Shimodaira H, Tokunaga S, Moriwaki T, Esaki T, Nagase M, Fujitani K, Yamaguchi K, Ura T, Hamamoto Y, Morita S, Okamoto I, Boku N, Hyodo I (2013). Randomized, open-label, phase III study comparing irinotecan with paclitaxel in patients with advanced gastric cancer without severe peritoneal metastasis after failure of prior combination chemotherapy using fluoropyrimidine plus platinum: WJOG 4007 trial. J Clin Oncol.

[6] Ford HE, Marshall A, Bridgewater JA, Janowitz T, Coxon FY, Wadsley J, Mansoor W, Fyfe D, Madhusudan S, Middleton GW, Swinson D, Falk S, Chau I, Cunningham D, Kareclas P, Cook N, Blazeby JM, Dunn JA (2014). COUGAR-02 Investigators. Docetaxel versus active symptom control for refractory oesophagogastric adenocarcinoma (COUGAR-02): an open-label, phase 3 randomised controlled trial. Lancet Oncol.

[7] Young K, Smyth E, Chau I (2015). Ramucirumab for advanced gastric cancer or gastro-oesophageal junction adenocarcinoma. Therap Adv Gastroenterol.

[8] Iacovelli R, Pietrantonio F, Farcomeni A, Maggi C, Palazzo A, Ricchini F (2014). Chemotherapy or targeted therapy as second-line treatment of advanced gastric cancer. A systematic review and meta-analysis of published studies. PLoS One.

[9] Shitara K, Doi T, Dvorkin M, Mansoor W, Arkenau HT, Prokharau A, Alsina M, Ghidini M, Faustino C, Gorbunova V, Zhavrid E, Nishikawa K, Hosokawa A, Yalçın Ş, Fujitani K, Beretta GD, Cutsem EV, Winkler RE, Makris L, Ilson DH, Tabernero J (2018). Trifluridine/tipiracil versus placebo in patients with heavily pretreated metastatic gastric cancer (TAGS): a randomised, double-blind, placebo-controlled, phase 3 trial. Lancet Oncol.

[10] Kang YK, Boku N, Satoh T, Ryu MH, Chao Y, Kato K, Chung HC, Chen JS, Muro K, Kang WK, Yeh KH, Yoshikawa T, Oh SC, Bai LY, Tamura T, Lee KW, Hamamoto Y, Kim JG, Chin K, Oh DY, Minashi K, Cho JY, Tsuda M, Chen LT (2017). Nivolumab in patients with advanced gastric or gastro-oesophageal junction cancer refractory to, or intolerant of, at least two previous chemotherapy regimens (ONO-4538-12, ATTRACTION-2): a randomised, double-blind, placebo-controlled, phase 3 trial. Lancet.

[11] Wilhelm SM, Dumas J, Adnane L, Lynch M, Carter CA, Schütz G, Thierauch KH, Zopf D (2011). Regorafenib (BAY 73–4506): a new oral multikinase inhibitor of angiogenic, stromal and oncogenic receptor tyrosine kinases with potent preclinical antitumor activity. Int J Cancer.

[12] Pavlakis N, Sjoquist KM, Martin AJ (2016). Regorafenib for the Treatment of Advanced Gastric Cancer (INTEGRATE): A Multinational Placebo-Controlled Phase II Trial. J Clin Oncol.

[13] Fuchs CS, Doi T, Jang RW, Muro K, Satoh T, Machado M, Sun W, Jalal SI, Shah MA, Metges JP, Garrido M, Golan T, Mandala M, Wainberg ZA, Catenacci DV, Ohtsu A, Shitara K, Geva R, Bleeker J, Ko AH, Ku G, Philip P, Enzinger PC, Bang YJ, Levitan D, Wang J, Rosales M, Dalal RP, Yoon HH (2018). Safety and Efficacy of Pembrolizumab Monotherapy in Patients With Previously Treated Advanced Gastric and Gastroesophageal Junction Cancer: Phase 2 Clinical KEYNOTE-059 Trial. JAMA Oncol.

[14] Janjigian YY, Bendell J, Calvo E, Kim JW, Ascierto PA, Sharma P, Ott PA, Peltola K, Jaeger D, Evans J, de Braud F, Chau I, Harbison CT, Dorange C, Tschaika M, Le DT (2018). CheckMate-032 Study: Efficacy and Safety of Nivolumab and Nivolumab Plus Ipilimumab in Patients With Metastatic Esophagogastric Cancer. J Clin Oncol.

[15] Janjigian YY, Shitara K, Moehler M, Garrido M, Salman P, Shen L, Wyrwicz L, Yamaguchi K, Skoczylas T, Campos Bragagnoli A, Liu T, Schenker M, Yanez P, Tehfe M, Kowalyszyn R, Karamouzis MV, Bruges R, Zander T, Pazo-Cid R, Hitre E, Feeney K, Cleary JM, Poulart V, Cullen D, Lei M, Xiao H, Kondo K, Li M, Ajani JA (2021). First-line nivolumab plus chemotherapy versus chemotherapy alone for advanced gastric, gastro-oesophageal junction, and oesophageal adenocarcinoma (CheckMate 649): a randomised, open-label, phase 3 trial. Lancet.

[16] Fukuoka S, Hara H, Takahashi N, Kojima T, Kawazoe A, Asayama M, Yoshii T, Kotani D, Tamura H, Mikamoto Y, Hirano N, Wakabayashi M, Nomura S, Sato A, Kuwata T, Togashi Y, Nishikawa H, Shitara K (2020). Regorafenib Plus Nivolumab in Patients With Advanced Gastric or Colorectal Cancer: An Open-Label, Dose-Escalation, and Dose-Expansion Phase Ib Trial (REGONIVO, EPOC1603). J Clin Oncol.

[17] Cancer Genome Atlas Research Network (2014). Comprehensive molecular profiling of lung adenocarcinoma. Nature.

[18] Martin AJ, Gibbs E, Sjoquist K, Pavlakis N, Simes J, Price T, Shannon J, Gill S, Jain V, Liu G, Kannourakis G, Kim YH, Kim JW, Goldstein D (2018). INTEGRATE I investigators. Health-related quality of life associated with regorafenib treatment in refractory advanced gastric adenocarcinoma. Gastric Cancer.

[19] Zheng Y, Zhu XQ, Ren XG. Third-line chemotherapy in advanced gastric cancer: a systematic review and meta-analysis. Medicine. 2017;96(24):e6884. 10.1097/MD.0000000000006884 PMC5478304 28614219

[20] El-Khoueiry AB, Kim RD, Harris WP, Sung MW, Waldschmidt D, Iqbal S, et al. Phase Ib study of regorafenib (REG) plus pembrolizumab (PEMBRO) for first-line treatment of advanced hepatocellular carcinoma (HCC). American Society of Clinical Oncology; 2020.

